# Damascenine induced hepatotoxicity and nephrotoxicity in mice and *in vitro* assessed human erythrocyte toxicity

**DOI:** 10.1515/intox-2015-0018

**Published:** 2015-09

**Authors:** Yacine Bouguezza, Bachra Khettal, Lydia Tir, Souad Boudrioua

**Affiliations:** Laboratoire de Biotechnologies végétales et Ethnobotanique, Faculté des Sciences de la Nature et de la Vie, Université de Bejaia, Bejaia 06000, Algeria

**Keywords:** Damascenine, hepatotoxicity, nephrotoxicity, erythrocyte, mice

## Abstract

*Nigella damascena* seed is characterized by the presence of the major alkaloid, damascenine and its related metabolites. To our knowledge, no detailed subchronic toxicological assessment of damascenine (DA) has been reported. The present study evaluated the potential toxicity of DA *in vivo* after sub-chronic intraperitoneal (i.p) administration in mice and *in vitro* following human erythrocyte hemolysis. *In vivo*, a total of 48 adult male and female Swiss albino mice were used in a sub-chronic toxicity study. The mice received intraperitoneally two doses of DA (20 and 100 mg/kg) for 28 days. Food intake, body weight and central body temperature were measured during the experiment. After completion of drug treatment, biochemical and histological analyses were performed. No mortality was observed in any of the treatment groups of mice, showing no toxic effects during the study. Neither were biochemical parameters altered; no significant differences were observed concerning glucose, bilirubin, aspartate transaminase (AST), alanine aminotransferase (ALT), urea, and creatinine parameters. No histopathological alterations were found in kidney and liver structures. *In vitro*, we focused on the human erythrocyte hemolytic process in the presence of several concentrations of DA. High level concentration of 1 000 μg/ml of DA revealed normal cell shapes and absence of hemolysis and deformation.

## Introduction

*Nigella damascena* is a herbaceous annual plant belonging to the Ranunculaceae family. It is used in Oriental herbal medicine for the treatment of catarrhal affections, amenorrhea and as a diuretic; powdered seeds are used as sternutator (Fico *et al.*, 2004). The seed of *Nigella damascena* is characterized by the presence of the major alkaloid, damascenine (3-methoxy-2-(methylamino) benzoic acid methyl ester) and its related metabolites were studied extensively by Döpke and Fritsch (1970). Damascenine is present in the seed to the extent of about 0.1–0.3 dry weight. The maximum amount of the alkaloid was found when the seed had reached maturity (Mohan *et al.*, 1965). Analgesic, antipyretic and antiedematous studies of damascenine were reported by Berkmeir *et al.* (1967). Acute toxicity was reported also by Berkmeir *et al.* (1967). In the latter study, the acute oral LD_50_ of damascenine in male mice amounted to 1 800 mg/kg and rats tolerated orally 1 600 mg/kg without any symptoms. Yet high intravenous (i.v) doses caused embolism of the lungs, while local irritation occurred after subcutaneous (s.c) injection.

Natural drugs are generally believed to be safer than chemical products, but some of them are harmful and may pose a clear and potent danger to human health. The safety of natural substances is constantly discussed and constitutes an important issue since the use of natural products is increasing worldwide (Vohra *et al.*, 2012 ; Necyk *et al.*, 2014). To our knowledge, no detailed toxicological assessment of DA has been reported. The purpose of our study was thus to screen subchronic hepatotoxicity and nephrotoxicity of DA in mice of both sexes and *in vitro* by assessing human erythrocyte hemolysis after acute exposure.

## Materials and methods

### Chemicals and reagents

Triton-X100, dimethyl sulfoxide (DMSO), petroleum ether (bp 30–40 °C), ammonium hydroxide solution (28% NH_3_ in H_2_O) and hydrochloric acid (37%) were obtained from Sigma-Aldrich Chemical (St. Louis, MO, USA). HPLC-grade acetonitrile was purchased from Merck Co. (Germany). Damascenine hydrochloride was purchased from ACC Corporation (USA).

### Plant material

Fresh seeds of *Nigella damascena* of one origin were imported from a commercial source from France. Seeds were authenticated by a botanist, prof. Benabdessalam from the Laboratory of Biotechnology and Ethnobotany at the University of Bejaia, Algeria. After washing and drying for 72 h at 35 °C, the seeds were ground using a Moulinex AR1044 grinder to obtain a fine powder and finally kept in tightly closed containers before extraction.

### Extraction of DA

The extraction of DA was performed according to the method reported by Ewin (1912). The powder of *Nigella damascena* seed (30 g) was wrapped in a thimble and placed in 250 ml Soxhlet extractor to be extracted first with petroleum ether for 8 h. The ether extract solution was evaporated using a rotary evaporator (Büchi 461, Germany) *in vacuum* at a reduced temperature of 40 °C to obtain fine muslin and combined with 5% hydrochloric solution. The solution was extracted with petroleum ether and the aqueous layer was made alkaline with ammonium and finally the alkaloid was extracted with petroleum ether to yield the total extract. The extract was dried and the ether removed by distillation under diminished pressure, leaving a residue which was distilled *in vacuum*. The yield of the extract (0.15%, w/w) was calculated with respect to the initial weight of the dry seed powder.

### Identification of DA by HPLC

The identification of DA was done according to Fico and Tomé (1998). The alkaloid extract was dissolved in acetonitrile and the analysis was performed using *Shimadzu Analytical HPLC* (Shimadzu, Japan) equipped with RP-C 18 and with an autosampler and UV-Vis detector column. MilliQ: CH_3_CN (3:7) (pH 8 with diethylamine) was used as eluent in isocratic conditions. The alkaloid extract was monitored at 230 nm.

### *In vitro* toxicity

#### Hemolysis study

Hemolysis assays were performed according to the method reported by Xian-guo and Ursula (1994) with minor modifications. Heparinized venous blood was obtained by venipuncture from healthy male and female volunteers. Serum and buffy coats were removed by centrifugation at 3 000 rpm for 10 min and the packed erythrocytes were washed three times in cold isotonic saline (150 mM, NaCl, pH=7). The suspensions of erythrocytes used in this study were freshly prepared daily. Sevenfold serial dilutions of DA were made in 10% DMSO (v:v) of isotonic saline. A total volume of 800 μl for each dilution of DA was placed in Eppendorf tubes. A vehicle tube (containing 10% of DMSO in isotonic saline) and a positive control tube (containing 1% Triton X-100 in isotonic saline) were also included in the analysis. All Eppendorf tubes were incubated at 37 °C for 30 min in a water bath. The reaction mixtures were centrifuged at 9 000×g for 5 min and the absorbance of the supernatants was measured at 415 nm using a UV-visible spectrophotometer (Analytikjena, SPECORD 50). The hemolytic rate was calculated in relation to hemolysis of erythrocytes in 1% Triton X-100, which was taken as 100%.

#### Evaluation of erythrocyte shape

For morphologic characterization, erythrocytes were exposed to DA for 2 h at final concentrations of 250 and 1 000 μg. 10 μl aliquots of erythrocytes were taken directly and the samples were mounted on a slide with a cover slip and the hemolysis was checked microscopically under an Optika light microscope (Optika, Italy) at (G×40) magnification to determine the presence or absence of intact erythrocytes.

### *In vivo* sub-chronic hepatotoxicity and nephrotoxicity studies

#### Experimental animals and housing conditions

All experiments were in compliance with the guidelines for the care and use of laboratory animals published by the US National Institute of Health (NIH publication No 85–23, revised 1985) with approval of the ethic committee of the pharmaceutical group company SAIDAL (Algiers, Algeria). Adult Swiss albino mice of both sexes weighing between 20 and 25 g, from the animal center of Pasteur's Institute (Algiers, Algeria) were used. The animals were randomly assigned into four groups and housed in transparent plastic cages (n=12; 6 males and 6 females separately) under standard laboratory conditions (ventilated room, 25±2 °C, 50±10% humidity, 12 h light/dark cycle from 06:00 h to 18:00h) and had free access to standard commercial diet (ONAB, Bejaia, Algeria) and tap water.

#### Subchronic toxicity study design

The experiment was conducted according to the protocols described by OECD Guideline 407 (OECD, 2008) with minor modifications. A total of 48 adult male and female Swiss albino mice were used in the sub-chronic toxicity study. They were randomly divided into 4 groups of 12 adult Swiss albino mice of both sexes (6 males and 6 females). The control group (Group 1) received normal saline solution (0.9% NaCl); the vehicle group (Group 2) received 10% of DMSO and two groups (Groups 3 and 4) received respective 20 mg/kg and 100 mg/kg DA (dissolved in 10% of DMSO). All treatments were administered by i.p. route in a volume of 1 mL/100 g body weight once daily for 28 days at 9:00 am. The body weight and central body temperature (anal) were monitored each week. Daily food intake of mice in each cage was measured by weighing the left-over food from the amount provided in the last 24 h.

### Histopathological study and blood biochemical analysis

The animals were fasted overnight and sacrificed by decapitation under anesthesia on the 29th day of the study. After blood collection, the kidney and liver were removed, cleaned with saline solution, weighed, and preserved in 10% formalin for histopathology examinations. Necropsy was conducted on all animals. The criteria of gross pathological examination were based on the position, shape, size, color, and consistency of the organs. Liver and kidney were divided into equal pieces using transverse portions (thickness 5 mm). The samples were then dehydrated in increasing ethanol concentrations (70 to 95%), cleared in xylene and embedded in paraffin wax. Histological sections of 4 μm thickness were performed using a Leica RM2025 rotary microtome. Sections were mounted on glass microscope slides, stained with hematoxylin and eosin (H&E). They were then examined by an Optika light microscope (Optika, Italy) for conventional morphological evaluation. The collected blood was centrifuged at 3 000 rpm for 10 min and serum was separated and assayed for glucose (GLU), aspartate amino transferase (AST), alanine amino transferase (ALT), total bilirubin (BIL), creatinine (CRE) and urea (URE) using a spin react biochemical kit (Spain).

### Statistical analysis

Comparisons among different groups were performed by analysis of variance (ANOVA). The differences among experimental and control groups were determined using the statistical software GraphPad Prism Ver.5.0 for Windows XP. Significant differences between control and experimental groups were assessed using one way ANOVA followed by Tukey's multiple comparison test. All data are expressed as mean ± standard error of measurement (S.E.M.); p-values less than 0.05 were considered to be significant.

## Results

### HPLC

**Figure 1:**
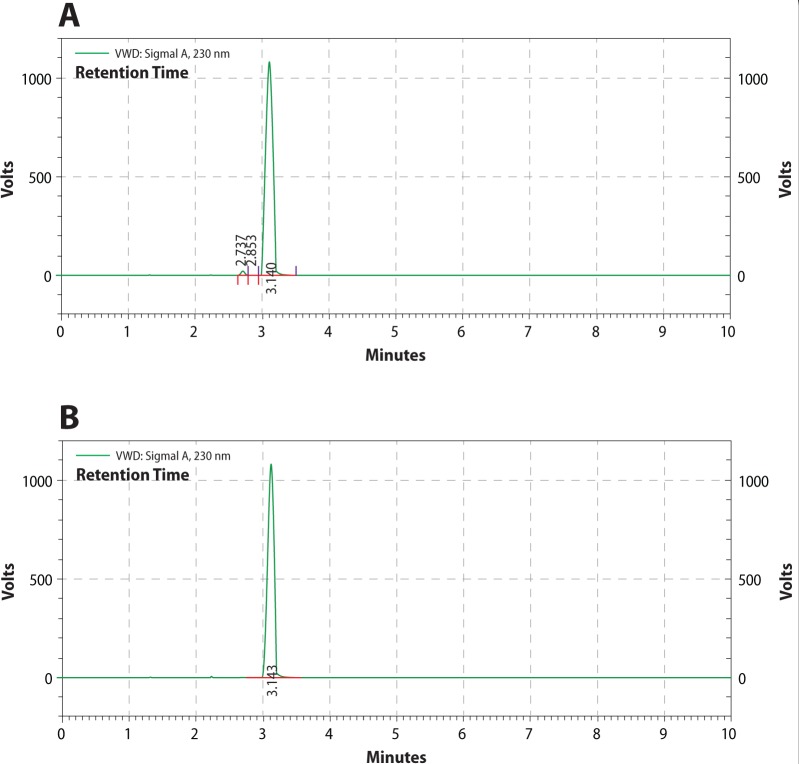
HPLC chromatogram of: (A) natural damascenine and its metabolites; (B) standard commercial damascenine.

A representative chromatogram is shown in Figure 1. The alkaloid extract was analyzed by HPLC. The results indicated that the alkaloid extract contained in the majority the alkaloid damascenine (about 95%, retention time of 3.140 min) and its related metabolites with a retention time of 2.737 and 2.853 min, respectively (Figure 1A). The major compound, damascenine, was identified by comparing its retention time with commercial damascenine under the same conditions (Figure 1B).

### *In vitro* hemolysis

**Figure 2:**
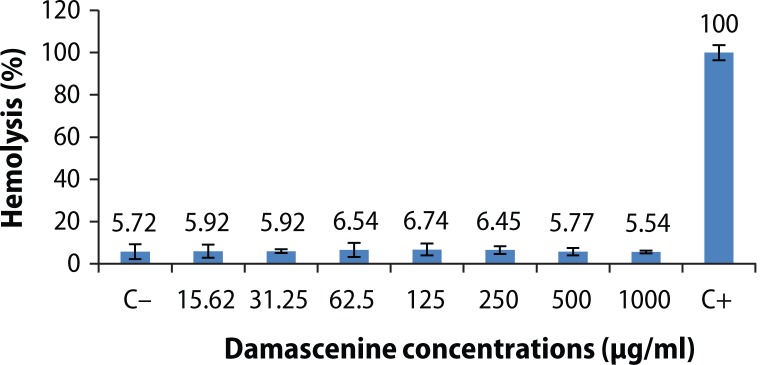
Hemolytic effect of damascenine. C^−^ (vehicle, negative control), C^+^ (1% Triton-X-100, positive control). Values are expressed as mean±SEM after three experiments. No significant difference (*p*>0.05) using one way ANOVA followed by Tukey’s multiple comparison test.

To test the cellular membrane disruptive effects of DA, human erythrocytes were incubated under isotonic conditions with DA (0–1000 μg/ml). Hemolysis was observed when the cells were incubated in the presence and absence of the alkaloid. The results (Figure 2) showed that DA had no hemolytic effect on erythrocytes.

### Evaluation of erythrocyte shape

**Figure 3:**
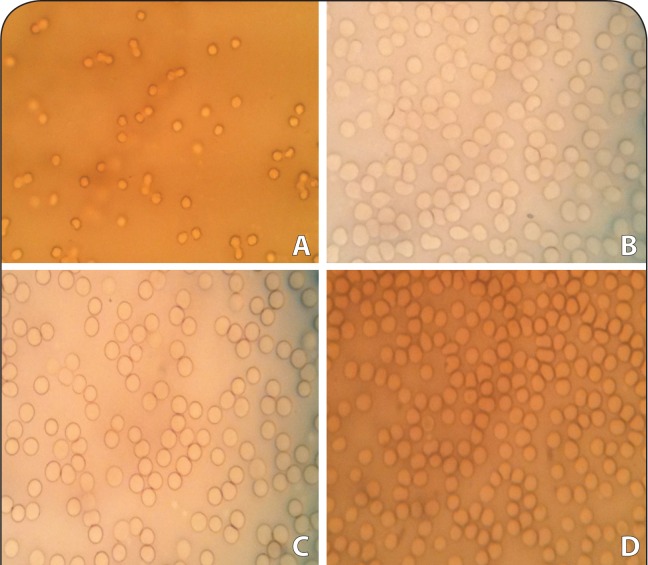
Photomicrographs (G×40) showing morphology of erythrocytes in the absence and presence of damascenine: (A) positive control cells incubated with 1% Triton-X100; (B) vehicle cells incubated with 10% DMSO; (C) cells incubated with 250 μg/ml of DA; (D) cells incubated with 1000 μg/ml of DA.

For the evaluation of shape changes in erythrocytes induced by DA, optical microscopy was performed. The results showed that the high concentration of 1 000 μg/ml of DA had no effect on cell shape (Figure 3). The cells incubated with 10% DMSO showed normal structure, yet those incubated with 1% Triton X-100 showed hemolysis and deformation.

### *In vivo* toxicity

#### Effect of DA on general behavior

No significant changes in general behavior or other major physiological activities of mice were observed at any time point in this study. No significant changes were recorded in body weight (Table 1), daily food intake (Table 2) and central body temperature (Table 3) in the treated mice as compared to controls. Both the control and treated mice appeared consistently healthy throughout the 28-day period of the study.

**Table 1:** Effects of damascenine on body weight of male and female Swiss albino mice.

	Day 1	Day 7	Day 14	Day 21	Day 28
**FEMALE**					
Saline	24.33±1.36	26.17±1.47	28.17±1.47	29.17±1.60	30.67±1.21
Vehicle	25.00±2.09	27.00±2.19	28.50±3.01	30.00±3.34	31.83±3.86
DA (20 mg/kg)	24.67±1.03	26.50±0.83	28.17±1.70	23.00±2.19	31.00±1.54
DA (100 mg/kg)	24.00±2.82	28.67±1.86	30.17±2.04	31.50±2.34	32.33±1.96
**MALE**					
Saline	25.00±1.26	26.83±1.32	30.00±1.41	29.17±1.60	34.33±1.75
Vehicle	25.67±0.81	28.33±0.81	32.17±1.83	34.67±2.25	35.33±2.87
DA (20 mg/kg)	25.00±1.09	28.83±1.94	32.50±2.88	35.33±3.14	35.50±3.98
DA (100 mg/kg)	24.67±2.42	28.00±2.44	32.50±2.58	36.17±3.25	35.83±4.21

Values are expressed as mean±SEM of 12 animals (6/sex). No significant difference using one way ANOVA, followed by Tukey’s multiple comparison test.

**Table 2:** Effects of damascenine on food intake of male and female Swiss albino mice.

	1st week (Day 1–7)	2nd week (Day 8–14)	3rd week (Day 15–21)	4th week (Day 22–28)
**FEMALE**				
Saline	34.43±3.10	32.43±4.07	29.14±2.03	29.29±1.70
Vehicle	26.29±1.11	26.86±3.23	25.43±2.37	26.86±0.89
DA (20 mg/kg)	30.71±1.89	28.43±4.03	26.29±1.49	26.71±1.49
DA (100 mg/kg)	32.43±3.82	30.71±2.62	26.43±2.14	27.14±1.46
**MALE**				
Saline	47.43±5.68	40.43±2.29	34.14±1.92	37.00±1.15
Vehicle	39.29±7.52	37.14±2.11	35.00±1.00	37.71±0.48
DA (20 mg/kg)	40.00±4.43	37.14±3.48	35.71±2.43	37.14±1.34
DA (100 mg/kg)	48.29±4.38	42.57±4.54	37.43±2.07	35.86±2.26

Values are expressed as mean±SEM of 12 animals (6/sex). No significant difference using one way ANOVA followed by Tukey’s multiple comparison test.

**Table 3:** Effects of damascenine on central body temperature of male and female Swiss albino mice.

	Day 1	Day 7	Day 14	Day 21	Day 28
**FEMALE**					
Saline	37.06±0.20	37.15±0.26	37.38±0.54	37.10±0.38	37.23±0.34
Vehicle	36.73±0.29	36.61±0.33	36.98±0.50	36.45±0.32	37.30±0.65
DA (20 mg/kg)	36.95±0.47	36.86±0.36	37.10±0.47	37.15±0.24	37.20±0.37
DA (100 mg/kg)	36.41±0.34	36.40±0.44	37.33±0.27	37.33±0.87	37.20±0.63
**MALE**					
Saline	37.26±0.37	37.23±0.67	38.65±0.18	38.03±0.32	36.65±0.36
Vehicle	37.28±0.45	37.40±0.43	38.15±0.62	37.91±0.86	37.01±0.37
DA (20 mg/kg)	37.23±0.78	37.35±0.84	37.78±0.70	37.95±0.70	37.28±0.41
DA (100 mg/kg)	37.46±0.39	37.60±0.49	37.90±0.26	37.96±0.84	37.80±0.30

Values are expressed as mean±SEM of 12 animals (6/sex). No significant difference using one way ANOVA followed by Tukey's multiple comparison test.

#### Effect of sub-chronic i.p. administration of DA on biochemical parameters of mice

The biochemical profiles of treated and control mice are presented in Table 4. Repeated i.p. administration of DA (daily dose of 20 and 100 mg/kg for 28 days) did not cause significant changes (*p*>0.05) in plasma creatinine, urea (kidney parameters), total bilirubin, glucose and the enzymes ALT and AST (liver parameters).

**Table 4:** Effects of damascenine on biochemical parameters of male and female Swiss albino mice.

Parameters	Control		Treatment (mg/kg)	
	Saline	Vehicle	20	100
ALT (U/L)	24.07±1.827	24.76±1.945	24.82±1.586	23.97±1.717
AST (U/L)	57.94±2.995	58.17±1.956	58.60±2.354	58.52±2.641
BIL (mg/L)	2.835±1.271	2.636±1.404	2.598±1.247	2.333±1.260
GLU (g/L)	1.208±0.310	1.153±0.335	1.323±0.273	1.266±0.316
CRE (mg/mL)	11.20±1.538	11.31±1.470	11.08±1.755	12.26±2.373
URE (g/L)	0.736±0.387	0.808±0.379	0.658±0.314	0.858±0.605

Values are expressed as mean±SEM of 12 animals (6/sex). No significant difference using one way ANOVA followed by Tukey’s multiple comparison test.

### Histopathological assessment of liver and kidney

#### Organ weight and morphology

The organ weights of different male and female groups treated with various dose levels of DA are shown in Table 5. Liver and kidney weight showed no morphological change in animals treated with 20 and 100 mg/kg in either the male or female groups. No significant changes (*p*>0.05) in kidney and liver weights of mice treated with DA were noticed when compared to the control group.

**Table 5:** Effects of damascenine on organ weight of male and female Swiss albino mice.

Organ weight (g)	Control		Treatment (mg/kg)	
	Saline	Vehicle	20	100
**LIVER**				
Female	1.397±0.219	1.488±0.241	1.553±0.183	1.413±0.217
Male	1.987±0.132	1.950±0.263	2.087±0.170	2.003±0.270
**KIDNEY**				
Female	0.371±0.044	0.395±0.079	0.356±0.031	0.376±0.045
Males	0.668±0.101	0.650±0.072	0.616±0.074	0.641±0.100

Values are expressed as mean±SEM of 12 animals (6/sex). No significant difference using one way ANOVA followed by Tukey’s multiple comparison test.

#### Liver and kidney tissue structure

**Figure 4:**
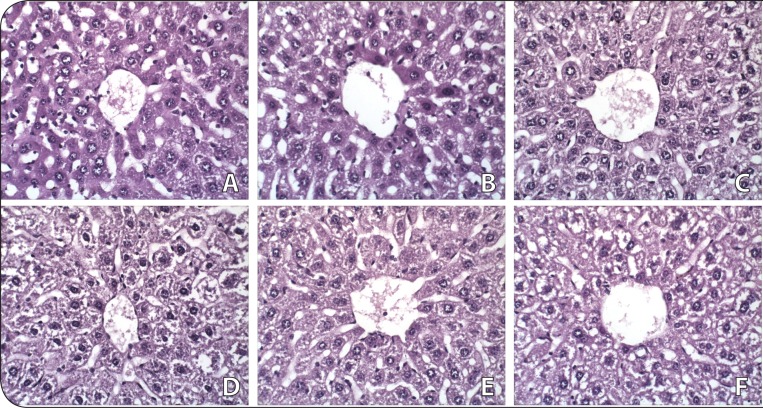
Photomicrographs (magnification: X40) of hematoxylin and eosin stained tissue sections taken from representative male and female Swiss Albinos mice treated with saline, vehicle and the highest dose of damascenine (100 mg/kg) on liver tissue during 28 days of treatment: Saline (A: males; B: females); Vehicle (10%DMSO) (C: males; D: females), 100 mg/kg of damascenine (E: males; F: females).

**Figure 5:**
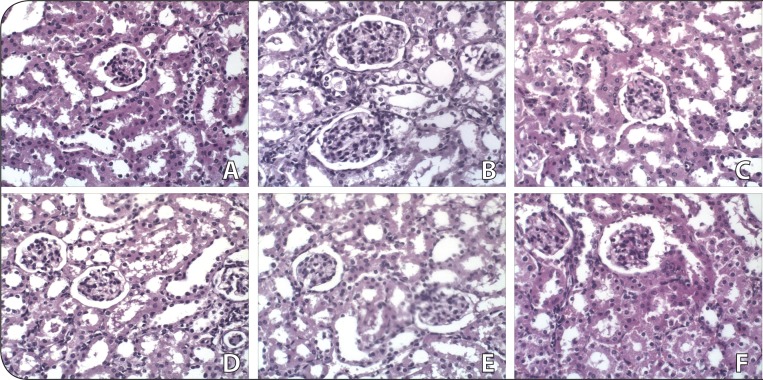
Photomicrographs (magnification: X40) of hematoxylin and eosin stained tissue sections taken from representative male and female Swiss Albino mice treated with saline, vehicle and the highest dose of damascenine (100 mg/kg) on kidney tissue during 28 days of treatment: Saline (A: males; B: females); Vehicle (10%DMSO) (C: males; D: females), 100 mg/kg of damascenine (E: males; F: females).

Figures 4 and 5 exhibit photomicrographs of liver and kidney structure; scale enlargement: ×40. The varying doses of DA (20 and 100 mg/kg) administered intraperitoneally to mice for 28 days did not induce any gross pathological lesion in liver and kidney cells of the test animals when compared to the control. The liver of all groups of mice showed normal parenchymal architecture with composed parenchymal cells or hepatocytes and non-parenchymal cells. The kidney of all groups showed a normal cortex and medulla region. Normal glomeruli with Bowman's capsule were observed. Endothelial cells were normal. The proximal and distal tubules were found to have normal and clear lumina.

## Discussion

The present study was aimed at evaluation of subchronic hepatotoxicity and nephrotoxicity of DA in Swiss albino mice and *in vitro* following human erythrocyte hemolysis. Exposure of Swiss albino mice to a daily single dose of 20 and 100 mg/kg (28 days) of DA did not produce any treatment-related effect. *In vivo*, various physical, biochemical and histological parameters were studied. The body weight and food intake were found to be unaltered during the 28-day treatment period when compared to the control group and the mice presented normal growth. No significant changes on liver and kidney weight were observed. The findings suggest no glossy toxic effect of DA at the sub-chronic *i.p* doses of 20 and 100 mg/kg. Central body temperature was found normal in comparison with the control group in both male and female mice, thus DA at the given doses had no effect on thermoregulation.

ALT and AST are present in high concentration in hepatocytes (Kew, 2000). Determination of ALT and AST in serum is a useful quantitative marker for the extent and type of hepatocellular damage (Udem *et al.*, 2009). AST is less specific than ALT as an indicator of liver function (Emerson *et al.*, 1993). Serum ALT activity level is the most frequently relied upon laboratory indicator of hepatotoxic effects, showing but infrequently false negative signals of liver histopathologic injury and limited false positive signals. It is considered the gold standard clinical chemistry marker of liver injury (Ozer *et al.*, 2008). The liver controls also glucose synthesis and generates free glucose from hepatic glycogen stores (Liangyou, 2014). In the present study, no significant changes were observed in ALT, AST, total bilirubin and glucose in treated animals when compared to controls (Table 4), suggesting that DA had no effects on liver functions of the mice at the given dose level.

Creatinine and urea are good renal markers for kidney function and the increase or decrease of these parameters reflects renal dysfunction (Sirwal *et al.*, 2004). In nephrology, the determination of plasma and urinary urea is exceeded by that of creatinine, but it remains a valuable marker (Valdiguié, 2000). In mammals, the highest concentration of creatinine found in skeletal muscle was associated with severe kidney damage (Wyss & Kaddurah-Daouk, 2000). Increase in bilirubin levels suggests increase in hemolysis intensity (Stillman, 1990).

No significant changes were observed in creatinine and blood urea in the treated animals when compared to controls (Table 4), suggesting that DA had no effect on kidney function at the doses given.

As shown in the histological analysis, no apparent lesions were observed in the kidney and liver (Figures 4 and 5). The kidney and liver are extremely sensitive to many chemical and environmental agents. The liver is involved in detoxification of xenobiotics by biotransformation (Edward & Celia, 1998). The kidney is responsible for elimination of unmodified drugs and metabolites and certain metabolites accumulated in the kidney may induce nephrotoxicity (Sener *et al.*, 2002). The hepatic and nephritic tissues showed normal architecture, which supported the biochemical and morphological analysis of these two organs.

*In vitro*, erythrocytes are readily available cells and a good model system to study the health status of individuals with pathologic complications. It can also serve as a meaningful target to study toxicant/xenobiotic induced damage (Bechan, 2010). Following hemolysis by hemoglobin liberated in the milieu after membrane disturbance, no significant differences between the cells in the presence and absence of DA were found and the high level concentration of 1 000 μg/ml of DA did not produce hemolysis, as confirmed by normal cell shape using microscopic visualization. Moreover, the acute and subchronic toxicity study of *Nigella damascena* methanolic seed extract in mice reported by Bouguezza *et al.* (2013) showed this extract to be safe.

In conclusion, the present investigation provides valuable information on the sub-chronic hepatoxicity and nephrotoxicity profiles of i.p. administration of DA and confirms the safety of *in vivo* experimental studies concerning the pharmacological potentialities of this mode of administration at the given level of drug dosage.

## References

[CIT0001] Bechan S, Devendra KR, Prashant KR, Rizvi SI, Geeta W (2010). Determination of erythrocyte fragility as a marker of pesticide-induced membrane oxidative damage. Methods Mol Biol.

[CIT0002] Bekemeier H, Leuschner G, Schmollack W (1967). Antipyretic, antiedematous and analgetic effects of damascenine in comparison with acetylsalicylic acid and phenylbutazone. Arch Int Pharmacodyn Ther.

[CIT0003] Bouguezza Y, Bribi N, Tacherfiout M, Amara S, Khettal B (2013). Acute and sub-chronic toxicity study of *Nigella damascena* methanolic seed extract in mice. Int J Pharm Bio Sci.

[CIT0004] Döpke W, Fritsch G (1970). Der Alkaloidgehalt von Nigella damascena L. Pharmazie.

[CIT0005] Edward AL, Celia JR (1998). Xenobiotic Metabolizing Enzymes of the Kidney. Toxicologic Pathology.

[CIT0006] Emerson FS, Shadara AC, Devi PU (1993). Toxic effects of crude extract of Plumbago rosea (Rokta chitraka). J Ethnopharmacol.

[CIT0007] Ewins AJ (1912). LXII. –The constitution and synthesis of damascenine, the alkaloid of Nigella damascena. J Chem Soc, Trans.

[CIT0008] Fico G, Tomè F (1998). Akaloids content in the ripening seeds of Nigella damascena. Europ J Pharmaceutical Sci.

[CIT0009] Fico G, Panizzi L, Flamini G, Braca A, Morelli I, Tomè F, Cioni PL (2004). Biological screening of Nigella damascena for antimicrobial and molluscicidal activities. Phytother Res.

[CIT0010] Kew MC (2000). Serum aminotransferase concentration as evidence of hepatocellular damage. Lancet.

[CIT0011] Liangyou R (2014). Energy metabolism in the liver. Compr Physiol.

[CIT0012] Mohan LV, Mothes K, Engelbrecht L, Schroter HB (1965). Biosynthesis of damascenine in Nigella damascena L. Nature.

[CIT0013] Necyk C, Tsuyuki RT, Boon H, Foster BC (2014). Pharmacy study of natural health product adverse reactions (SONAR): a cross-sectional study using active surveillance in community pharmacies to detect adverse events associated with natural health products and assess causality. BMJ Open.

[CIT0014] OECD, Test guideline 407 (2008). Repeated dose oral toxicity test method. Organization for Economic Cooperation and Development.

[CIT0015] Ozer J, Ratner M, Shaw M, Bailey W, Schomaker S (2008). The current state of serum biomarkers of hepatotoxicity. Toxicology.

[CIT0016] Sener G, Sehirli AO, Altunbas H.Z, Ersoy Y, Paskaloglu KA, Ayanoglu-Dugler G (2002). Melatonin protects against gentamicin induced nephrotoxicity in rats. J Pineal Res.

[CIT0017] Sirwal IA, Banday KA, Reshi AR, Bhat MA, Wani MM (2004). Estimation of Glomerular Filteration Rate (GFR). JK Science.

[CIT0018] Stillman AE, Walker HK, Hall WD, Hurst JW (1990). Jaundice, in *Clinical Methods*: The History, Physical, and Laboratory Examinations.

[CIT0019] Udem SC, Obidoa O, Asuzu IU (2009). Acute and chronic toxicity studies of Erythrina senegalensis DC stem bark extract in mice. Comp Clin Pathol.

[CIT0020] Valdiguié P (2000). Constituant azotés non protéique. Biochimie Clinique (2*^ème^*ed Mé*dicales Internationales*).

[CIT0021] Vohra S, Cvijovic K, Boon H, Foster BC (2012). Study of natural health product adverse reactions (SONAR): active surveillance of adverse events following concurrent natural health product and prescription drug use in community pharmacies. PLOS ONE.

[CIT0022] Wyss M, Kaddurah-Daouk R (2000). Creatine and creatinine metabolism. Physiol Rev.

[CIT0023] Xian-guo HE, Ursula M (1994). Antifungal compound from Solanum nigrescens. J Ethnopharmacol.

